# Flu DRiPs in MHC Class I Immunosurveillance

**DOI:** 10.1007/s12250-018-0061-y

**Published:** 2018-11-19

**Authors:** Jiajie Wei, Jonathan W. Yewdell

**Affiliations:** Cellular Biology Section, Laboratory of Viral Diseases, National Institute of Allergy and Infectious Diseases, National Institutes of Health, Bethesda, MD 20892 USA

**Keywords:** Influenza A virus (IAV), MHC-restricted cytotoxicity, Immune responses, MHC class I, DRiP

## Abstract

Since the publication of the DRiP (defective ribosomal product) hypothesis in 1996, numerous studies have addressed the contribution of DRiPs to generating viral antigenic peptides for CD8^+^ T cell immunosurveillance. Here, we review studies characterizing the generation of antigenic peptides from influenza A virus encoded DRiPs, discuss the many remaining mysteries regarding the nature of their co-translational generation, and speculate on where the future might lead.

## Introduction

Influenza A virus (IAV), a negative-sense, single-stranded, segmented RNA virus, causes significant worldwide morbidity, mortality and economic burden due to its evasion of adaptive immunity, despite repeated infection and vaccination. CD8^+^ T cell immunosurveillance of MHC class I-peptide complexes, a crucial part of the adaptive immune system, recognizes peptides encoded by each of the eight IAV gene segments, limits viral replication and reduces morbidity and mortality in hosts whose antibody responses fail to prevent infection (McMichael *et al.*^1983^; Kim *et al.*^2011^). Vaccines that induce and boost CD8^+^ T cells are promising candidates for improving the duration of effective immunity to IAV (Souquette and Thomas 2018), since many of the immunogenic IAV peptides are highly conserved among human IAV isolates.

By binding and presenting oligopeptides at the cell surface, MHC class I molecules provide a window into the translational status of cells. This enables CD8^+^ T cell recognition of viruses and other intracellular pathogens, cancers, transplants and autoimmune targets. MHC class I molecules also function in other biological processes, including neutral killer cell activation, mate selection, and neuronal development (McAllister 2014; Apanius *et al.*^2017^).

Viruses have played a central role in understanding the MHC class I biology, starting from the discovery of MHC restriction, which provided the first clear evolutionary justification for a system discovered based on its prominent role in graft rejection (Zinkernagel and Doherty 1974). CD8^+^ T cells play an important role in the clearance of many viruses. The sheer number and variety of viral proteins known to interfere with various steps of the class I pathway underscores the importance of antigen presentation in viral immunity and viral evolution (Schuren *et al.*^2016^).

MHC class I antigenic peptides encoded by host or viral genes have two potential sources: “retirees” (Yewdell 2001, 2003) and DRiPs (defective ribosomal products) (Yewdell *et al.*^1996^). Retirees are proteins that reach stable structures and degrade with normal turnover kinetics, *i.e.* a median half-life of 46 h across the entire proteome (Schwanhäusser *et al.*^2011^). DRiPs, a substantial subset of nascent gene products that degrade more rapidly than their corresponding native retiree pools (Wheatley *et al.*^1980^; Schubert *et al.*^2000^; Wang *et al.*^2013^), were originally proposed to explain the rapidity of CD8^+^ T cell recognition of virus infected cells. Viral peptide ligands can be generated well within an hour after viruses are added to cells (Esquivel *et al.*^1992^; Croft *et al.*^2013^; Zanker *et al.*^2013^).

DRiPs were originally conceived as misfolded or prematurely terminated proteins arising as inevitable errors in protein biogenesis and errors deliberated enhanced by innate cellular responses to infection (Yewdell *et al.*^1996^). The DRiP hypothesis has evolved over the years (Yewdell 2003, 2011; Yewdell and Nicchitta 2006; Anton and Yewdell 2014) to encompass all possible errors that can occur in converting genetic information into mature proteins, including stoichiometric excess subunits of multiprotein complexes (Bourdetsky *et al.*^2014^), and products of non-canonical translation and mistranslation. Importantly, numerous studies support that immunological relevant viral peptides predominately originate from DRiPs, including products from downstream initiation on AUG codons (Berglund *et al.*^2007^), frame shifting (Fetten *et al.*^1991^; Bullock and Eisenlohr 1996; Elliott *et al.*^1996^; Zook *et al.*^2006^), initiation on non-AUG start codons (Yang *et al.*^2016^), stop codon read through, and translation of viral RNA in the nucleus (Dolan *et al.*2010a).

## Flu DRiPs

The first studies demonstrating that peptides can efficiently arise from rapidly degraded defective proteins came from Alain Townsend’s pioneering research showing that CD8^+^ T cells lyse cells expressing rapidly degraded versions of truncated influenza proteins expressed from transgenes (Townsend *et al.*^1985^, ^1986^). These findings were extended by Fetten *et al.* (1991) who found that presentation of a NP peptide to CD8^+^ T cells continued apace after introduction of an upstream frameshift that completely abrogated detection of full length NP encoded by a retrovirus transduced gene. These findings were extended to mutated IAV proteins expressed by recombinant vaccinia viruses (Bullock and Eisenlohr 1996; Elliott *et al.*^1996^), which became a workhorse for mechanistic antigen processing studies due to the relative ease of generating recombinant viruses that could be used to infect many cells lines *in vitro* as well as animals *in vivo*.

With time, it became possible to use IAV itself to study the effects of genetic alterations on class I peptide generation (Webby *et al.*^2003^). This was an essential step to understanding IAV peptide generation, since the rules of generating peptides are likely to vary depending on the exact nature of how the protein is synthesized, which varies considerably among different virus familes. For example, we found that the same protein encoded by a vaccinia virus generated mRNA *vs.* a host cell transgene encoded-mRNA generates peptide with different efficiencies per retiree molecule degraded (Dolan *et al.*^2012^). Still, the small coding capacity of IAV sets a fairly tight limit on adding genetic information and the need for the virus to replicate limits genetic manipulation of essential genes.

The engineering of the model H2-K^b^ binding 8-mer SIINFEKL peptide into IAV (Jenkins *et al.*^2006^), in combination with K^b^-SIIINFEKL detection reagents B3Z hybridoma cells (Karttunen *et al.*^1992^), OT-I TCR transgenic mice (Clarke *et al.*^2000^), and the 25-D1.16 monoclonal antibody (Porgador *et al.*^1997^), provides an invaluable system for sensitively and quantitatively studying peptide generation *in vitro* and in mice. To study natural IAV DRiPs, Dolan *et al.* genetically inserted SIINFEKL into the stalk of IAV neuraminidase (NA-SIINFEKL) and monitored K^b^-SIINFEKL presentation on cell surface after IAV infection (Dolan *et al.*2010b). As far as known, the only function of the NA stalk is to set the height of NA globular domain, and stalk length varies widely among various circulating NA genes in the animal reservoir. Indeed, SIINFEKL insertion did not change NA translation, folding, degradation, transport, or surface expression. Kinetic analysis using 25-D1.16 for flow cytometric determination of K^b^-SIINFEKL cell surface generation revealed that K^b^-SIINFEKL is generated in lockstep with initiation and abrogation of NA synthesis in both L-K^b^ fibroblast cells and DC2.4 dendritic/monocyte cells, supporting the importance of natural viral DRiPs during infection.

Dolan *et al.* (2010a) reported an intriguing disconnect between standard NA-SIINFEKL translation and K^b^-SIINFEKL generation. Unlike most RNA viruses, IAV (and other myxoviruses) express their mRNA in the nucleus, where the mRNAs steal caps from cellular mRNA to enable their export from the nucleus and translation. RNA polymerase (RNAP) II inhibitors, 5,6-dichloro-1-b-D-ribofuranosylbenzimidazole (DRB) and actinomycin D, prevent nuclear of export some IAV mRNAs (Amorim *et al.*^2007^), including NA, as Dolan *et al.* reported. Remarkably, a 33-fold reduction of NA expression in DRB treated cells was accompanied by only a 5-fold decrease in K^b^-SIINFEKL cell surface expression. This is consistent with the idea that translation of NA-SIINFEKL RNA in the *nucleus* accounts for a fraction of antigenic peptide generation from NA.

Nuclear translation is a highly contentious topic (Dahlberg *et al.*^2003^; Iborra *et al.*^2004^; David *et al.*^2012^; Reid and Nicchitta 2012; Al-Jubran *et al.*^2013^), and its resurrection stirred the hornet’s nest (Dahlberg and Lund 2012). Its possible contribution to immunosurveillance was further supported by studies showing that OT-I T cells equally recognize cells expressing SIINFEKL from introns *vs.* exons in transfected genes (Apcher *et al.*^2013^). K^b^-SIINFEKL generation from both exons and intronic SIINFEKL was unaffected by blocking mRNA export from the nucleus, suggesting nuclear translation of prespliced RNA as a surprising source for peptide generation.

Over several decades the Shastri lab pioneered studies demonstrating the usage of non-canonical translation start sites for MHC class I peptide immunosurveillance. With the Pan lab, they discovered that antigenic peptides can be initiated from CUG with an elongator leucine-tRNA rather than the canonical AUG start codon with Met-tRNA (Starck *et al.*^2012^). This translation is based on initiation factor eIF2 rather than canonical eIF2A initiation (Starck *et al.*^2016^). Yang *et al.* (2016) extended these findings to IAV immunosurveillance by studying the generation of K^b^-SIINFEKL complexes from the IAV *M2* gene with SIINFEKL at its C-terminus. IAV exploits its nuclear transcription to create a number of spliced mRNAs. Gene segment seven encodes two proteins: M1 is translated from unspliced mRNA while all but the 9-N-terminal residues in M2 encoded by the M1 +1 reading frame from a spliced mRNA. Yang found that while the mRNA splicing inhibitor spliceostatin A nearly completely inhibited M2 mRNA generation and protein synthesis, K^b^-SIINFEKL complexes were still robustly generated. The use of CUG codons in unspliced M1 mRNA as initiation codons was supported by three lines of evidence. First, K^b^-SIINFEKL was generated *in vitro* and *in vivo* from mRNA synthesized in the cytoplasm by vaccinia virus, whose mRNAs are not known to be subject to splicing, which is thought to only occur in the nucleus. Second, drugs that block AUG initiation did not reduce the level of K^b^-SIINFEKL complexes. Third, and most conclusively, synonymous mutation of CUG codons severely reduced K^b^-SIINFEKL generation. Thus, Yang *et al.* defined an IAV DRiP generated by cytoplasmic noncanonical translation, and demonstrates the participation of CUG-codon–based translation initiation in pathogen immunosurveillance.

Even more remarkably, although the negative stranded genomic RNA of IAV is nearly “non-coding” by definition, Hickman *et al.* (2018) found that K^b^-SIINFEKL complexes can be generated from two different negative strand gene segments. A long open reading frame that is highly conserved among human and animal IAVs is present in the genomic strand of segment eight, encoding a potential ~167 residue protein termed NEG8 (Zhirnov *et al.*^2007^; Clifford *et al.*^2009^). Despite a lack of biochemical evidence of NEG8 protein expression, K^b^-SIINFEKL complexes are generated when SIINFEKL is appended to the predicated COOH-terminus of NEG8, since infection with the recombinant virus activates OT-I T cells *in vitro* and *in vivo*. SIINFEKL embedded in the negative strand of the NA-stalk coding sequence also activates OT-I T cells *in vivo,* albeit weakly. These findings demonstrate translation of the IAV negative strand can also contribute to DRiPs generation and anti-viral immunosurveillance. The fascinating questions of where in the cell (nucleus *vs.* cytoplasm) and how the IAV negative stand is translated remain to be investigated.

## IAV DRiPomics

A central aspect of the DRiPs theory holds that DRiPs are an inevitable result of protein translation. Although the biochemical nature of DRiPs generated from viral proteins remains a work very much in progress, mass spectrometry-based studies that correlate viral protein synthesis with peptide generation provide definitive evidence for DRiPs as a major source of viral peptides. In a groundbreaking study, Croft *et al*. (2013) used a novel approach, liquid chromatography coupled with a targeted variation of mass spectrometry termed multiple reaction monitoring, to quantify eight vaccinia virus immunogenic peptides and their source proteins at multiple time points after infecting cultured cells. This revealed a tight correlation between the onset of protein expression and peptide presentation, providing the strongest biochemical evidence to date for the central contribution of DRiPs to presentation of viral peptides during acute cell infection.

Wu extended this approach to IAV (2017), identifying 22 IAV-derived class I bound peptides including eight previously unknown peptides. Peptides bound by surface MHC-I on IAV infected DC2.4 cells were then quantified across a time course of infection with the abundance of viral source proteins measured simultaneously. Importantly, nearly all of the viral peptides were detected prior to or simultaneously with their source proteins, clearly demonstrating the general relevance of DRiPs to IAV immunosurveillance.

## Future Directions

The next step in defining the contribution of DRiPs to viral immunosurveillance is to use ribosome profiling (Ribo-Seq) to identify all possible translation events. In RiboSeq, RNase resistant ribosome protected fragments are deep sequenced in conjunction with the use of translation inhibitors to enable identification of initiation, stalling, and termination events (Ingolia *et al.*^2011^, ^2014^). The frequency of translation is inferred by read numbers of translated sequences. Despite the power and progressive optimization of RiboSeq (McGlincy and Ingolia 2017), the method is challenging both technically and informatically.

While information from RiboSeq will be essential in unraveling the mysteries in DRiP generation and will likely identify additional immunogenic IAV peptides, other approaches will also be important. The efficiency of generating class I binding peptides from DRiPs can differ several fold from highly similar virus-encoded substrates, pointing to important differences in exactly how proteins are delivered to proteasomes for degradation (Princiotta *et al.*^2003^). Further, intracellular peptide competition studies showed that while preprocessed peptides competed for class I presentation as expected from the law of mass action, they are unable to inhibit presentation by peptides liberated from DRiPs (Lev *et al.*^2010^), consistent with the idea of compartmentalized translation, degradation, and delivery of peptides to the TAP. Further, the burgeoning heterogeneity in ribosome structures (Slavov *et al.*^2015^; Shi *et al.*^2017^; Genuth and Barna 2018), ribosomes themselves are likely to have modifications that modulate their ability to generate antigenic peptides (Yewdell and Nicchitta 2006; Wei and Yewdell 2018).

Adding to the complexity and interest, a recent report concludes that 30% of class I peptides are generated by peptide splicing (Liepe *et al.*^2016^), presumably via the proteasome (Hanada *et al.*^2004^; Vigneron *et al.*^2004^; Warren *et al.*^2006^). As proteasome-mediated splicing is an uncommon, though potentially efficient event using purified proteasomes (Berkers *et al.*^2015^), this points to the possibility of special treatment of spliced peptides by the class I pathway. It will certainly be of great interest to explore the contribution of peptide splicing to viral immunosurveillance.

Finally, it is highly likely that tissue and cell type specific viral epitopes exist and are exploited by the immune system. *In vivo* studies that combine RiboSeq and mass spec of MHC-I peptides, although technically challenging given the large amount of starting material needed, are absolutely needed to validate and extend findings with cultured cells.

## Working Together to Improve Global Health

This review was written to commemorate the highly successful visit of NIH virologists in March 2018 to Wuhan and Beijing to meet Chinese virologists and share their expertise and knowledge. Among all pathogens, viruses pose the greatest risk to humanity due to their ubiquity and ability to rapidly mutate. Viruses do not respect political boundaries between countries. With the ever expanding human population, increased travel of individuals between once isolated locales, and man-made rapid changes in climate that alter local ecologies, the risk for catastrophic viral pandemics constantly increases. Increased communication between virologists, including organized mechanisms for sharing data, reagents and people, will be essential to minimize the impact of viruses on human health.

On a personal note, one of the joys as a senior scientist (JWY), is to work with talented and enthusiastic young scientists from around the globe. In the course of my career at NIH, I have had the great pleasure of working with seven post-docs from China, all of whom made important discoveries in my laboratory. I thank the people of China for raising and training such outstanding people, and to urge that the scientific ties between our countries, and all countries, continue to increase.

The fate of the world depends on it!
